# *N*-acylethanolamide metabolizing enzymes are upregulated in human neural progenitor-derived neurons exposed to sub-lethal oxidative stress

**DOI:** 10.3389/fncel.2022.902278

**Published:** 2022-08-08

**Authors:** R. Scott Duncan, Sean M. Riordan, Conner W. Hall, Andrew J. Payne, Kent D. Chapman, Peter Koulen

**Affiliations:** ^1^Department of Ophthalmology, Vision Research Center, School of Medicine, University of Missouri–Kansas City, Kansas City, MO, United States; ^2^Department of Biological Sciences, Center for Plant Lipid Research, University of North Texas, Denton, TX, United States; ^3^Department of Biomedical Sciences, School of Medicine, University of Missouri–Kansas City, Kansas City, MO, United States

**Keywords:** cannabinoid, neurons, neuroprotection, *N*-acylethanolamide, oxidative stress, transcription factor

## Abstract

*N*-acyl amides (NAAs) are a class of lipids that consist of an acyl group N-linked to an amino acid, neurotransmitter, taurine or ethanolamide group (*N*-acylethanolamines or NAEs) and include some endocannabinoids (eCB) such as anandamide. These lipids are synthesized in a wide variety of organisms and in multiple cell types, including neurons. NAEs are involved in numerous cellular and physiological processes and their concentrations are elevated in response to ischemia and physical trauma to play a role in neuroprotection. The neuroprotective properties of eCB NAEs make the protein targets of these compounds attractive targets for clinical intervention for a variety of conditions. The most promising of these targets include cannabinoid receptor type 1 (CB1), cannabinoid receptor type 2 (CB2), fatty acid amide hydrolase (FAAH), *N*-acylethanolamine acid amidase (NAAA), and *N*-acyl phosphatidylethanolamine phospholipase D (NAPE-PLD). Further characterization of these targets in a more contemporary model system of neurodegeneration and neuroprotection will allow us to fully describe their role and mechanism of action in neuroprotection against oxidative stress leading to better utilization in the clinical setting. Human stem cell-derived or human neural progenitor cell-derived cells, such as ReN cells, have become more utilized for the study of human neuronal development and neurodegenerative diseases. ReN cells can be easily differentiated thereby circumventing the need for using transformed cell lines and primary neurons as cell model systems. In this study, we determined whether ReN cells, a superior cell model system for studying neurodevelopment, differentiation, and neuroprotection, express proteins involved in canonical eCB NAE signaling and whether oxidative stress can induce their expression. We determined that sublethal oxidative stress upregulates the expression of all eCB proteins tested. In addition, we determined that oxidative stress increases the nuclear localization of FAAH, and to a lesser extent, NAAA and NAPE-PLD. This study is a first step toward determining how oxidative stress affects CB1, CB2, FAAH, NAAA, and NAPE-PLD expression and their potential defense against oxidative stress. As such, our data is important for further determining the role of eCB metabolizing proteins and eCB receptors against oxidative stress.

## Introduction

There is ample evidence demonstrating the cytoprotective effects of endogenous cannabinoids (eCBs) and non-eCB *N*-acylethanolamides (NAEs). For example, *N*-acyl phosphatidylethanolamine (NAPE) and NAE synthesis is upregulated in response to multiple chemical and traumatic environmental insults, underscoring their role in cytoprotection (Natarajan et al., 1986; Hansen et al., 1995, 1997, 1998; Moesgaard et al., 1999, 2000; Hansen et al., 2001; Schabitz et al., 2002; Berger et al., 2004).

eCBs have been shown to play a role in alleviating multiple diseases including cardiovascular disease (Kunos et al., 2000; Steffens et al., 2005), liver dysfunction (Julien et al., 2005; Teixeira-Clerc et al., 2006; Jeong et al., 2008; Jourdan et al., 2010), intestinal inflammation (Izzo et al., 2001; Massa et al., 2004), peripheral and neuropathic pain (Siegling et al., 2001; Lim et al., 2003; Zhang et al., 2003), and neurodegeneration (Skaper et al., 1996; Hansen et al., 2002; Marsicano et al., 2003; Kim et al., 2005; Van der Stelt and DiMarzo, 2005; Shouman et al., 2006; Yiangou et al., 2006; Wang et al., 2007).

The neuroprotective NAE, anandamide or arachidonoylethanolamide (AEA), and the non-NAE endocannabinoid, 2-arachidonyl glycerol (2-AG), are the prototypical eCBs as they can bind to and activate cannabinoid receptor type 1 (CB1) (Devane et al., 1992; Vogel et al., 1993). In addition to CB1, 2-AG can also activate cannabinoid receptor type 2 (CB2). The NAE, palmitoylethanolamide (PEA), functions as a decoy eCB NAE to inhibit the degradation of AEA by the NAE-degrading enzyme, fatty acid amide hydrolase (FAAH) (Jonsson et al., 2001; Smart et al., 2002). Other proteins involved in eCB signaling include monoacylglycerol lipase (MAGL), the saturated NAE degrading enzyme *N*-acylethanolamide acid amidase (NAAA) and the NAE synthesizing enzyme *N*-acylethanolamide specific phospholipase D (NAPE-PLD) (Matsuda et al., 1990; Kaminski et al., 1992; Munro et al., 1993; Di Marzo et al., 1996; Ueda et al., 2000; Petersen et al., 2009).

Several studies have determined that cannabinoid compounds, or overexpression of components of the endocannabinoid system, can protect cells from oxidative stress (Elmazoglu et al., 2020). Much less is known, however, about whether oxidative stress can alter the expression and activity of endocannabinoid proteins. Superoxide anion can induce the expression of CB1 (Wang et al., 2014). CB1 has been sown to be upregulated in Parkinson’s Disease patients and postmortem brain from AD patients (Brotchie, 2003; Aso and Ferrer, 2014), both diseases where oxidative stress plays an important pathophysiological role. Some redox-sensitive transcription factors, such as AP-1, Nrf2, NFκB, and STATs, can upregulate the expression of proteins involved in eCB signaling, including CB1 and FAAH (Maccarrone et al., 2003a,b; Börner et al., 2008; Hsu et al., 2018; Galán-Ganga et al., 2020; Hay et al., 2020) although most of these effects were not experimentally observed under oxidative stress conditions.

Human stem cell-derived or human neural progenitor cell-derived cells, such as ReN cells, are becoming more commonly used for the study of human neuronal development and neurodegenerative diseases. These cells can be kept in an undifferentiated proliferative state, and they can easily be differentiated by removing two growth factors from the culture media. This quality circumvents the need for isolating primary neurons from brain tissue and it allows for a considerable amount of cellular material that can be used in several large experiments.

Here, we determined whether chemical induction of oxidative stress in ReN cells leads to alteration, particularly upregulation of eCB protein expression. We determined that ReN cells express CB1, CB2, FAAH, NAAA, and NAPE-PLD and that oxidative stress leads to an increase in the immunoreactivity of these proteins. As 2-AG is not an NAE eCB, and the 2-AG degrading MAGL does not degrade NAE eCBS, MAGL was not included in this study. These results suggest that oxidative stress may elicit the upregulation of eCB proteins as part of their response to oxidative damage.

## Materials and methods

### Cell culture and treatments

Human neural precursor-derived ReN cells were obtained from Millipore (Millipore-Sigma, Burlington, MA, United States) and were cultured on Matrigel^®^ in ReN neural stem cell (NSC) maintenance media (Millipore-Sigma) supplemented with 10 ng/ml EGF (Gibco/ThermoFisher Scientific, Waltham, MA, United States) and 10 ng/ml bFGF (Gibco). Cells were grown on Matrigel^®^ -coated surfaces in an undifferentiated state in the presence of bFGF and EGF until ready for use in experiments. ReN cells were differentiated by maintaining cells in ReN NSC maintenance media without EGF of bFGF for at least 7 days and up to 14 days. ReN cells on coverslips were treated with tert-butylhydroperoxide (tBHP) (Acros Organics, Geel, Belgium) at a concentration of 2 or 10 μM overnight (16–20 h) prior to fixation with 4% PFA and subsequent immunocytochemistry.

### Antibodies, immunocytochemistry, and immunofluorescence

Rabbit anti CB1 (ab23703), CB2 (ab3561), FAAH (ab128917), and rabbit and mouse anti-NAPE-PLD (ab246951 and ab119259) were purchased from Abcam. NAAA antibodies were purchased from EzBiolab (Westfield, IN, United States). Antibodies used in this study except for NAAA have been characterized and used by others (Lowin et al., 2015; Aichler et al., 2017). Immunocytochemistry and immunofluorescence were conducted as described elsewhere (Duncan et al., 2007). In brief, ReN cells were grown on Matrigel coated 12 mm glass coverslips for 7–10 days to a density of ≤75% confluence. Fixation of cells was carried out using 4% paraformaldehyde for 15 min followed by washing with PBS. Primary antibody incubation was carried out at 4°C overnight. After washing with PBS, fluorescent Alexa488 or Alexa594-labeled goat anti-rabbit or goat anti-mouse IgG secondary antibody (Invitrogen) diluted in incubation solution (1:1,000) was applied to cells for 1 h at room temperature followed by 3 washes in PBS. Coverslips were mounted onto glass slides with Aqua polymount (Polysciences, Warrington, PA, United States) and stored at 4°C overnight. Image acquisition was conducted with a Leica TCS SP5 X white light laser scanning confocal microscope (Leica, Buffalo Grove, IL, United States) using 20X, 40X, or 63X objectives. LAS-X software was used to collect 8 or 16-bit TIFF images.

Western blots were carried out using a standard SDS-PAGE mini gel system (ThermoFisher-Life Technologies, Carlsbad, CA, United States) with 7% Bis-Tris SDS-PAGE gels and nitrocellulose membranes. Ponceau-S dye was used to stain membranes for relative total protein measurement used for normalization. Proteins were visualized using Luminata Forte chemiluminescent solution (Millipore-Sigma). Membranes were visualized using a G:box gel/membrane imaging system (Syngene, Bangalore, India).

### Image analysis

Brightfield images were visualized and acquired using a Leica DMIL inverted objective microscope under phase contrast settings. Resulting brightfield images were used to determine cell number and neurite density in Image J FIJI software (NIH; free open source). Neurite density was determined by converting images to a binary (black and white) image and tracing lines from the top to the bottom of the image being careful to avoid soma. The number of peaks caused by intersecting a line were then counted and graphed. Microfluorimetric analysis was conducted on TIFF images using Image-J FIJI. To determine levels of immunoreactivity in images, images were converted to maximum intensity projections and were then thresholded to exclude background signal and to identify regions where mean fluorescence intensity, fluorescence area, and fluorescence integrated density could be determined. Cell (nuclear) counts were either calculated using manual counts or by using the 3D Cell Counter setting. Colocalization analysis was done in Image J using the Coloc2 function which compares the intensity of each pixel from one channel of a thresholded image to the intensity of each pixel in another channel of a thresholded image. Quantitative data is presented as a Pearson’s, Spearman’s, or Mander’s coefficient (*R*^2^ value). Data was exported into Microsoft Excel and graphed. Average fluorescence intensity measurements were done using threshold settings established for maximum intensity projection images. Densitometric analysis of Western blot images was carried out by measuring average intensity and integrated density of bands using Image-J FIJI. Immunoblot data was normalized to Ponceau-S total protein.

### Groups and statistics

For all microfluorimetry experiments, five (5) microscope fields per treatment group from three different experiments were analyzed for CB1, CB2, FAAH, NAAA, and NAPE-PLD. For Western blots, densitometry was carried out on results from three separate experiments. Treatment groups included untreated control, 2 or 10 μM tBHP. An *F*-test was conducted in Excel between each treatment group and the untreated control group to determine which type of *T*-test should be used for group comparisons–*T*-test with equal or unequal variance. The mean fluorescence intensity from each treatment group was separately compared to the mean fluorescence intensity of the untreated control group using a two-sample *T*-test with either equal or unequal variances. Comparison between more than two groups was done in GraphPad Prism^®^ software using a one-way ANOVA test with Bonferroni *post*-*hoc* analysis to determine which groups are different from controls.

## Results

### Establishing sublethal oxidative stress conditions in ReN cells

**FIGURE 1:**
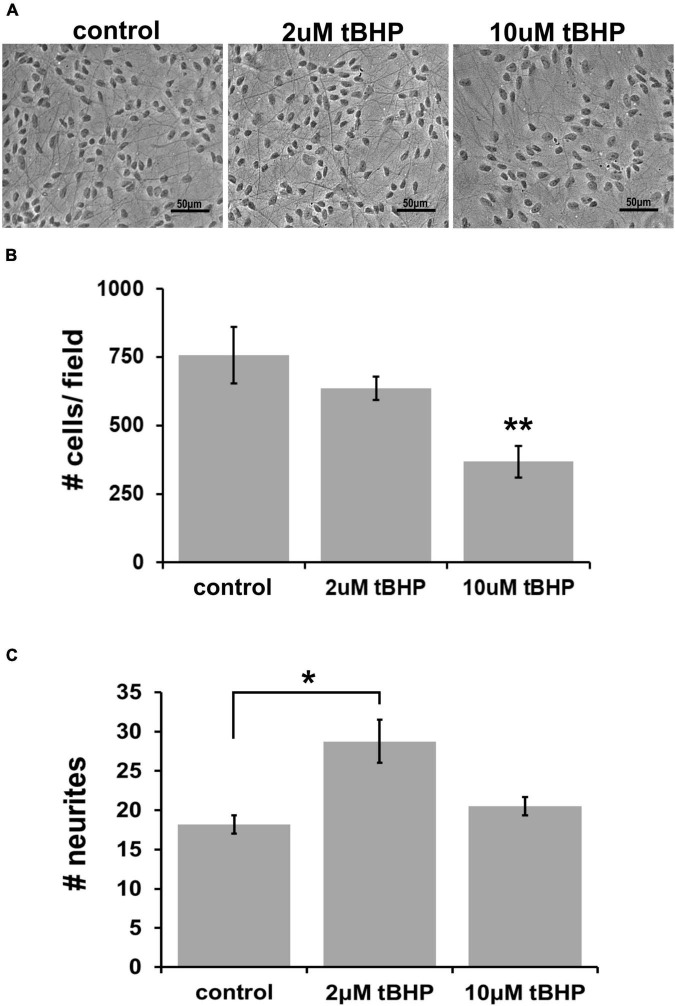
Establishing the optimal parameters for oxidative stress in ReN cells. **(A)** Phase contrast image showing differentiated ReN cells under control conditions (cont) or exposure to 2 μM or 10 μM tert-butylhydroperoxide (tBHP). Note that there is a reduction in the number of cells when exposed to 10 μM tBHP (right panel). Magnification is 200×, scalebar is 50 μM. **(B)** Manual cell counts from phase contrast images reveal that 2 μM tBHP led to a 16% reduction while 10 μM tBHP led to a 52% reduction in the number of cells. **(C)** Measurement of the number of neurites along an intersecting line from the top of the field to the bottom of the field while avoiding soma reveals that 2 μM tBHP led to a 45% increase in neurite density while 10 μM tBHP had no effect on neurite density. Images were converted to binary (black and white) and lines were drawn from the top to the bottom of the field. The number of times the intensity went from 255 to 0 (encounter with a neurite) was counted and recorded to determine the number of neurites. Neurites from > 10 cells (between 10 and 20 cells) were analyzed and three samples, each with six trials, were measured to determine neurite number. *p ≤ 0.05, **p ≤ 0.01.

Previous oxidative stress and neuroprotection studies have determined that endocannabinoids can protect against a highly lethal concentration of tBHP up to 25 μM (Duncan et al., 2009). To establish the adequate concentration of tBHP to elicit a much milder level of oxidative stress (sublethal oxidative stress), we tested two tBHP concentrations (2 and 10 μM) that we had previously established to induced sublethal oxidative stress in a murine hippocampal cell line, HT-22 (Kaja et al., 2010). We exposed ReN cells to 2 and 10 μM tBHP (and vehicle control) for 24 h and, after fixing, we acquired images of cells with a light microscope using phase contrast filters (Figure 1A).

Exposure of cells to 2 μM tBHP led to a 16% reduction in the number of cells (636 ± 25 cells vs. 757 ± 60 cells) while 10 μM led to a 52% reduction in the average cell number (Figure 1B). The remaining attached cells exposed to either 2 or 10 μM tBHP had normal size and cellular morphology that suggested that they were healthy. Cells exposed to 10 μM tBHP tended to exhibit larger nuclei. We measured the number of neurites that intersected a line drawn across images of cells. The cells exposed to 2 μM tBHP had a higher number of neurites (45% increase) on average than cells from the control condition. The cells exposed to 10 μM tBHP exhibited no change in the number of intersecting neuritis (Figure 1C). Together, these data suggest that the milder oxidative stress (2 μM tBHP) causes slight cell loss but an increase in the density of neurites while the higher level of oxidative stress (10 μM tBHP) led to a greater degree of cell loss but a lower density of neurites. We conclude that 2 and 10 μM are adequate concentrations of tBHP to determine the effect of oxidative stress on eCB protein expression.

### Immunofluorescent detection of cannabinoid receptor type 1 and cannabinoid receptor type 2 in ReN cells under conditions of oxidative stress

**FIGURE 2:**
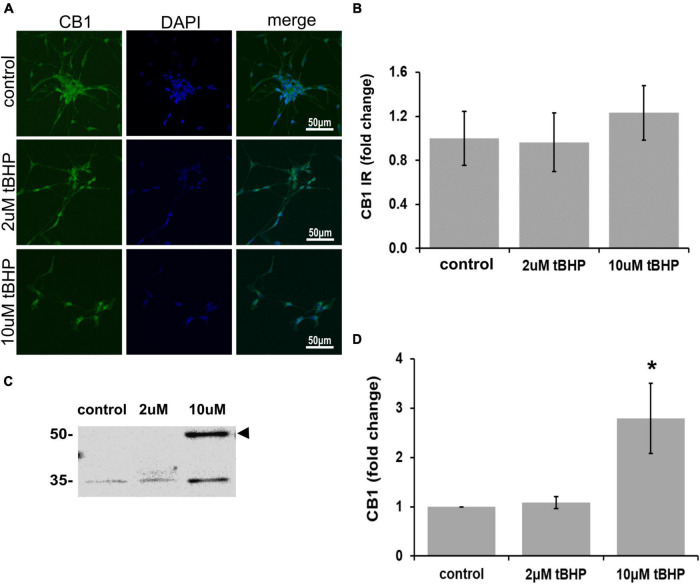
Expression of cannabinoid receptor type 1 (CB1) in ReN cells under conditions of oxidative stress. CB1 immunoreactivity in differentiated ReN cells. **(A)** Images show that tert-butylhydroperoxide (tBHP) has no effect on CB1 IR (green). Green fluorescence is CB1 and blue fluorescence is DAPI-labeled nuclei. Images are 400× magnification. **(B)** Quantitative analysis of images in panel **(A)** reveal that tBHP has no significant effect on CB1 IR, but a trend toward an increase exists (0.10 > *p* > 0.05). **(C,D)** Western blot analysis reveals that the expression of a 56 kDa band corresponding to CB1 is upregulated 2.8-fold by exposure to 10 μM tBHP. *p ≤ 0.05.

CB1 has been shown to be important for neuronal development, differentiation, neurotransmission, and viability (Hansen et al., 1995, 2001). Other studies on CB1 and oxidative stress have shown that CB1 activation can lead to neuroprotection (Skaper et al., 1996; Hansen et al., 2002). ReN cells express CB1 in the soma and neurites and treatment with tBHP to induce sublethal oxidative stress had no effect on CB1 immunoreactivity by microscopic methods (IR; fluorescence integrated density) (Figures 2A,B). However, immunoblotting revealed a 2.8-fold increase in CB1 following 10 μM tBHP treatment (Figures 2C,D). Slight inconsistencies between the immunofluorescence and Western blot data are likely due to the native vs. denatured form of the target protein, respectively. We consider trends in the same direction (up or down) as corroborating results.

**FIGURE 3:**
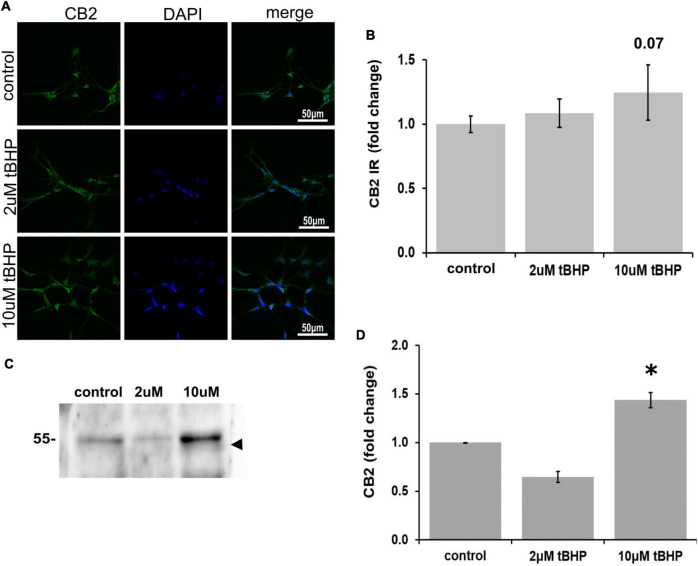
Expression of cannabinoid receptor type 2 (CB2) in ReN cells under conditions of oxidative stress. **(A)** Images show that tert-butylhydroperoxide (tBHP) has no apparent effect on CB2 IR (green). Blue are DAPI-labeled nuclei. Images are 400× magnification. **(B)** Quantitative analysis of images in panel **(A)** reveals that tBHP has no significant effect on CB2 IR, although a trend toward an increase exists (0.10 < *p* < 0.05). **(C,D)** Western blot analysis reveals that the expression of a 48 kDa band (expected molecular weight of glycosylated CB2 is 46 kDa) corresponding to CB2 is downregulated by 40% by exposure to 2 μM tBHP and upregulated 45% by exposure to 10 μM tBHP. *p ≤ 0.05.

There has been considerable debate over recent decades as to whether CB2 is expressed in neurons or only in non-neuronal cells such as glia (Van Sickle et al., 2005; Onaivi et al., 2006; Liu et al., 2009), because of this uncertainty we investigated whether ReN cells express CB2. ReN cells express CB2 in both the soma and the neurites (Figure 3A). Treatment with tBHP to induce sublethal oxidative stress had no significant effect on CB2 expression by microscopy/immunofluorescence methods, but trended toward an increase in fluorescence integrated density (Figures 3A,B). Western blotting revealed that 2 μM tBHP led to a 40% decrease in CB2 while exposure to 10 μM tBHP led to a 45% increase in CB2 expression in ReN cells (Figures 3C,D), suggesting that CB2 gene expression is regulated by oxidative stress.

### Immunofluorescent detection of fatty acid amide hydrolase in ReN cells under conditions of oxidative stress

**FIGURE 4:**
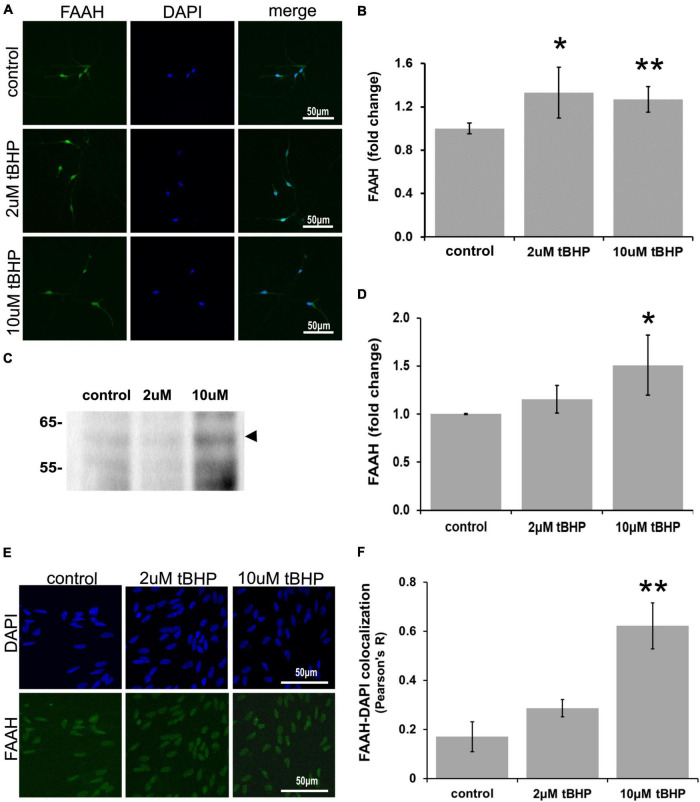
Expression of fatty acid amide hydrolase (FAAH) in ReN cells under conditions of oxidative stress. Exposure of ReN cells to sublethal concentrations of tert-butylhydroperoxide (tBHP) led to an increase in FAAH expression **(A,B)**. Green fluorescence is FAAH and blue fluorescence is DAPI. **(C,D)** Western blot analysis reveals that the expression of a 63 kDa band corresponding to FAAH is unaffected by exposure to 2 μM tBHP but upregulated 1.5-fold by 10 μM tBHP. The expected molecular weight of FAAH is 63 kDa. **(E,F)** Colocalization analysis between FAAH immunoreactivity with DAPI fluorescence (DNA) reveals low colocalization at resting untreated conditions (*R* = 0.18), but 2 μM tBHP exposure led to a 68% increase in Pearson’s *R* (from 0.17 to 0.28). Higher tBHP concentrations (10 μM) led to a large (3.6-fold) increase in FAAH-DAPI colocalization (from *R* of 0.17 to 0.62), suggesting significant FAAH localization in the nucleus near DNA. *p ≤ 0.05, **p ≤ 0.01.

ReN cells express FAAH predominantly in the soma, especially the nucleus (Figure 4). Since FAAH is important for the regulation of intracellular NAE levels, changes in its expression level may be important for the amplitude and duration of eCB action. Exposure of ReN cells to tBHP led to an increase in FAAH immunoreactivity (Figures 4A,B), suggesting that eCB degradation by FAAH may be increased and therefore less available for signaling at CB1 or CB2 receptors. FAAH is expressed at high levels in the nuclear envelope or nucleus as determined by immunofluorescence (Figure 4A). Others have observed this nuclear localization as well (Chamley et al., 2008). Immunoblotting revealed that exposure to 10 μM tBHP led to a 1.5-fold increase in FAAH expression (Figures 4C,D).

To determine if oxidative stress selectively alters the nuclear localization of FAAH, we carried out colocalization analysis between FAAH immunoreactivity and DAPI nuclear stain fluorescence. We reasoned that if FAAH was expressed on the nuclear envelope, there would likely not be much colocalization. However, if FAAH was expressed inside the nucleus, there should be some colocalization with the DAPI DNA stain if the proximity between the two is within a few hundred nanometers. We define no colocalization as an *R*^2^ value below 0.15, weak colocalization as an *R*^2^ value between 0.16 and 0.25, moderate colocalization as an *R*^2^ value between 0.26 and 0.50 and strong colocalization as an *R*^2^ value of ≥0.51.

Exposure of cells to 2 μM tBHP led to a 68% (1.7-fold) increase in FAAH-DAPI colocalization (Pearson’s correlation coefficient *R*^2^ = 0.17 ± 0.06 for the untreated control vs. 0.29 ± 0.04 for 2 μM tBHP) (Figures 4E,F). Exposure of cells to 10 μM tBHP led to a 3.6-fold increase in FAAH-DAPI colocalization compared to untreated controls and 2.1-fold increase in FAAH-DAPI colocalization compared to 2 μM tBHP (Pearson’s correlation coefficient *R*^2^ = 0.17 ± 0.06 for the untreated control vs. 0.29 ± 0.04 for 2 μM tBHP vs. 0.63 ± 0.09 for 10 μM tBHP).

Because 10 μM tBHP exposure caused some cell death (Figure 1B), remaining nuclei may exhibit higher FAAH-DAPI co-localization due to nuclear condensation from oxidative damage. We measured the average nuclear area and determined that ReN cells exposed to 10 μM tBHP had a larger than average nucleus compared to nuclei from untreated and 2 μM tBHP-treated ReN cells. This suggests that the increase in FAAH-DAPI colocalization is not due to nuclear condensation but is due to actual colocalization of FAAH and DAPI (DNA).

### Immunofluorescent detection of *N*-acylethanolamine acid amidase in ReN cells under conditions of oxidative stress

**FIGURE 5:**
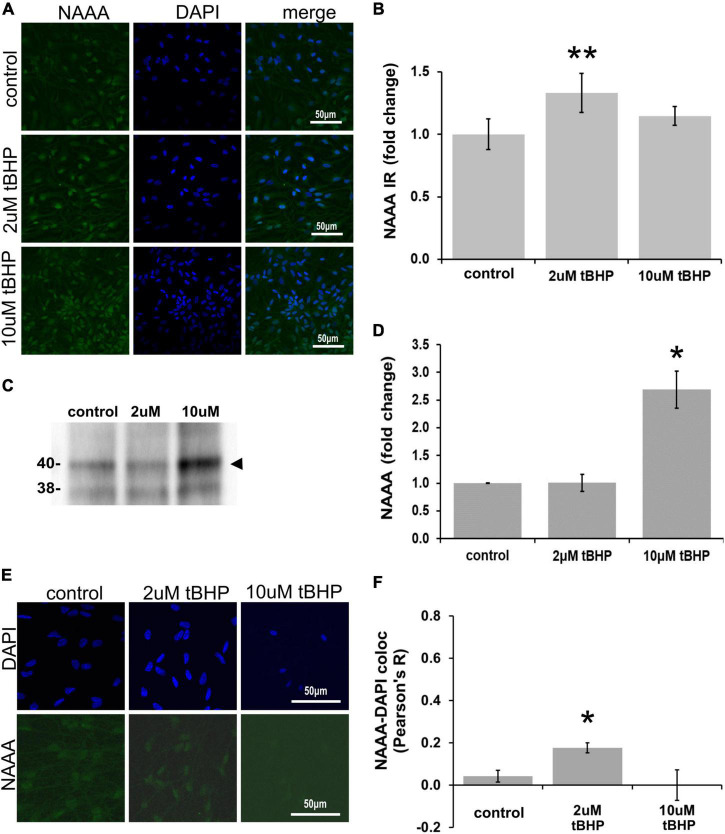
Expression of *N*-acylethanolamine acid amidase (NAAA) in ReN cells under conditions of oxidative stress. **(A)** ReN cells express NAAA in the soma, including in the nuclear envelop and possibly in the nucleus, as well as neurites. **(B)** Exposure of ReN cells to sublethal concentration of tert-butylhydroperoxide (tBHP) resulted in a biphasic NAAA expression pattern–2 μM tbHP led to a 35% increase in NAAA immunoreactivity while 10 μM did not, although a trend toward an increase existed. Green fluorescence is NAAA and blue fluorescence is DAPI. **(C,D)** Western blot analysis reveals that the expression of a 40-kDa band (expected molecular weight is 40 kDa) corresponding to NAAA is upregulated 2.6-fold by exposure to 10 μM tBHP by. **(E,F)** Colocalization analysis between NAAA immunoreactivity with DAPI fluorescence (DNA) reveals no significant colocalization at resting untreated conditions, but 2 μM tBHP exposure led to a slight increase in Pearson’s *R* (from 0.03 to 0.20). Higher tBHP concentrations (10 μM) had no effect on NAAA-DAPI colocalization. *p ≤ 0.05, **p ≤ 0.01.

ReN cells express NAAA in the soma (non-nuclear and nuclear) and neurites (Figures 5A,B). The exposure of ReN cells to 2 μM tBHP led to a 30% increase in NAAA immunoreactivity and a return to control levels at a tBHP concentration of 10 μM (Figure 5B). Western blot analyses showed that 2 μM tBHP had no effect on NAAA expression while 10 μM caused a 2.6-fold increase in NAAA expression (Figures 5C,D). This suggests that eCB degradation by NAAA is induced by oxidative stress.

To determine if oxidative stress selectively alters nuclear NAAA, we carried out colocalization analysis between NAAA IR and DAPI nuclear stain fluorescence (Figures 5E,F). NAAA did not colocalize with DAPI (*R*^2^ = 0.042) like FAAH, suggesting that apparent nuclear localization of NAAA may be due to NAAA expression in the nuclear envelope and not inside the nucleus. Exposure of ReN cells to 2 μM tBHP led to a 4.2-fold increase in NAAA-DAPI colocalization compared to untreated controls (Pearson’s correlation coefficient *R*^2^ = 0.18 ± 0.024 for 2 μM tBHP vs. 0.042 ± 0.028 for the untreated controls) (Figures 5E,F). Despite this multi-fold increase, it is still considered a weak level of colocalization. Exposure of cells to 10 μM tBHP led to a reversal in NAAA-DAPI colocalization compared to 2 μM tBHP (Pearson’s correlation coefficient *R*^2^ = < 0.00001 ± 0.07 for 10 μM tBHP vs. 0.18 ± 0.024 for 2 μM tBHP and 0.042 ± 0.028 for untreated) and failed to colocalize at all with DAPI. Together, these results suggest that, unlike FAAH, NAAA subcellular localization is not within the nucleus and the increase in NAAA colocalization with DAPI due to 2 μM tBHP is very low.

### Immunofluorescent detection of *N*-acyl phosphatidylethanolamine phospholipase D in ReN cells under conditions of oxidative stress

**FIGURE 6:**
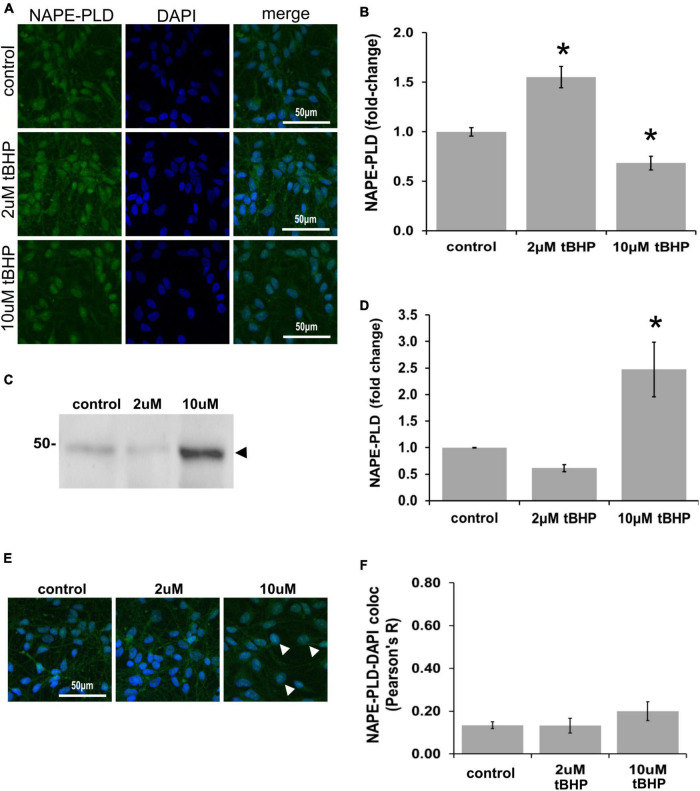
Expression of *N*-acyl phosphatidylethanolamine phospholipase D (NAPE-PLD) in ReN cells under conditions of oxidative stress. **(A)** ReN cells express NAPE-PLD in the soma and nucleus. **(B)** Exposure of ReN cells to tert-butylhydroperoxide (tBHP) resulted in a biphasic NAPE-PLD expression pattern–2 μM tBHP led to a 20% increase in NAPE-PLD expression. Green fluorescence is NAPE-PLD and blue fluorescence is DAPI. **(C,D)** Western blot analysis reveals that the expression of a 48 kDa band (expected molecular weight is 45.6 kDa) corresponding to NAPE-PLD is upregulated 2.4-fold by exposure to 10 μM tBHP. **(E,F)** Colocalization analysis on image stacks reveals that there is little colocalization between NAPE-PLD and DAPI (Pearson’s *R* value < 0.20) except for when cells are exposed to 10 μM tBHP, a concentration that increases colocalization to Pearson’s *R* of 0.20. *p ≤ 0.05.

*N*-acyl phosphatidylethanolamine phospholipase D (NAPE-PLD) immunoreactivity in ReN cells is increased by 18% in response to tBHP exposure, but failed to reach statistical significance (Figures 6A,B). NAPE-PLD immunoreactivity was predominately located in the soma and nucleus. Western blot analysis, however, revealed that exposure of ReN cells to 10 μM tBHP led to a 2.4-fold increase in NAPE-PLD expression (Figures 6C,D). Colocalization analysis determined whether NAPE-PLD was located in the nucleus of ReN cells. Colocalization analysis revealed that in untreated cells and cells treated with low concentration of tBHP (2 μM tBHP), there is little colocalization between NAPE-PLD and DAPI (Pearson’s R value < 0.20) (Figures 6E,F) Cells treated with a higher tBHP concentration (10 μM tBHP), however, showed slightly higher colocalization (Pearson’s R^2^ = 0.20 for 10 μM tBHP vs. 0.13 for untreated controls) (Figures 6E,F). This suggests that NAPE-PLD is not localized within the nucleus, and oxidative stress only slightly increase its nuclear localization.

## Discussion

In this study, we determined whether oxidative stress induced the expression of eCB proteins by using NPC derived neuronal model of neuronal differentiation, neurodegeneration, and neuroprotection. Human ReN cells may be more relevant for the *in vitro* study human diseases than primary mouse or rat cell models. We determined that ReN cells express the NAE eCB proteins CB1, CB2, NAPE-PLD, FAAH, and NAAA and that oxidative stress leads to an increase in their immunoreactivity. Other proteins involved in eCB signaling, such as MAGL and diacylglycerol kinase A (DAGK), were excluded from this study as they are not involved in NAE eCB signaling. To our knowledge, there are no studies that have determined whether oxidative stresses can upregulate the expression of NAPE-PLD in neuronal cells. Overall, these results identify potential molecular targets for pharmacological interventions against neurodegenerative diseases where oxidative stress and damage may play a major role.

There is evidence that redox-sensitive transcription factors can regulate the expression of CB1, CB2, and FAAH genes. T cell activation leads to an upregulation in CB1 expression *via* AP-1, NFκB, and NFAT (Börner et al., 2008) and AP-1 activation in hippocampal cells upregulated CB1 expression (Hay et al., 2020). In kidney cells, CB1 upregulation by indoxyl sulfate occurs *via* ATF3/c-Jun. The CB2 gene promoter contains Nrf2 (Nfe2l2) sites that are critical for CB2 expression in microglia (Galán-Ganga et al., 2020). Furthermore, oxidative stress upregulates the expression of CB1 and CB2, and downregulates the expression of FAAH, in RPE cells (Wei et al., 2009). These studies, however, did not utilize a model for oxidative stress.

Using immunofluorescence to detect NAE signaling proteins, it was possible to detect FAAH, NAAA, and NAPE-PLD proteins in or around the nucleus. This nuclear localization may indicate that NAE synthesis and degradation near the nucleus may affect nuclear signaling and/or activation of lipid-interacting transcription factors (i.e., PPARα and PPARγ). More research in this area is needed, however, to determine whether this is the case.

We observe some differences in the quantitative changes in proteins between data from immunofluorescence and Western blot densitometry analyses. While it is ideal to see a similar degree of change in protein expression between the two methods, the methods differ in both the type of antibody used (i.e., monoclonal vs. polyclonal) and/or structural nature of the protein epitope (native structure in ICC/IF and denatured structure in Western blotting). As a result, one might expect some differences in the magnitude of change between two different methods. Also, the measurement of the signal between fluorescence (in IF) and chemiluminescence (in Westerns) is different as the former exhibits a curvilinear relationship and the later a linear relationship between the measured signal and actual protein concentration. Our view, therefore, is that if the overall signal between the IF and Western blotting goes in the same direction (i.e., up or down) resulting from one of the two tBHP concentrations, then the methods satisfactorily corroborate one another, especially given the different nature of the two methods.

These data provide relevant information about both cannabinoid and non-cannabinoid proteins in ReN cells. Several diseases are beginning trials for or is already utilizing stem cells transplantation strategies as a therapy including retinal degeneration, ischemic stroke and myocardial infarction (Cehajic-Kapetanovic et al., 2022; Mitreèiæ et al., 2022; Zhao et al., 2022). Since the endocannabinoid system is known to be involved in early cellular and tissue development and cell fate, better understanding the role of eCB proteins, such as CB1, CB2, FAAH, and others, may be important in establishing better cell transplant outcomes.

## Data availability statement

The original contributions presented in this study are included in the article, further inquiries can be directed to the corresponding author.

## Author contributions

PK conceived and designed the experiments. RD, SR, AP, and CH performed the experiments. RD and PK wrote the manuscript. All authors analyzed the data, edited and reviewed the manuscript, and approved the submitted version.
